# Efficacy of virtual reality exposure therapy for treatment of dental phobia: a randomized control trial

**DOI:** 10.1186/s12903-016-0186-z

**Published:** 2016-02-27

**Authors:** Kumar Raghav, AJ Van Wijk, Fawzia Abdullah, Md. Nurul Islam, Marc Bernatchez, Ad De Jongh

**Affiliations:** Department of Behavioural Sciences, Academic Centre for Dentistry Amsterdam (ACTA), University of Amsterdam and VU University, 1081 LA Amsterdam, The Netherlands; Faculty of Dentistry, SEGi University, No:9 Jalan Teknologi, Kotadamansara, PJU-5, Petalingjaya-47810, Selangor, Malaysia; Virtual Simulations Inc, Quebec, Canada; School of Health Sciences of Salford University, Manchester, UK

**Keywords:** Dental phobia, Virtual reality exposure therapy

## Abstract

**Background:**

Virtual Reality Exposure Therapy (VRET) is found to be a promising and a viable alternative for in vivo exposure in the treatment of specific phobias. However, its usefulness for treating dental phobia is unexplored. The aims of the present study are to determine: (a) the efficacy of VRET versus informational pamphlet (IP) control group in terms of dental trait and state anxiety reductions at 1 week, 3 months and 6 months follow-up (b) the real-time physiological arousal [heart rate (HR)] of VRET group participants during and following therapy (c) the relation between subjective (presence) and objective (HR) measures during VRET.

**Methods:**

This study is a single blind, randomized controlled trial with two parallel arms in which participants will be allocated to VRET or IP with a ratio of 1:1. Thirty participants (18-50 years) meeting the Phobia Checklist criteria of dental phobia will undergo block randomization with allocation concealment. The primary outcome measures include participants’ dental trait anxiety (Modified Dental Anxiety Scale and Dental Fear Survey) and state anxiety (Visual Analogue Scale) measured at baseline (T0), at intervention (T1), 1-week (T2), 3 months (T3) and 6 months (T4) follow-up. A behavior test will be conducted before and after the intervention. The secondary outcome measures are real-time evaluation of HR and VR (Virtual Reality) experience (presence, realism, nausea) during and following the VRET intervention respectively. The data will be analyzed using intention-to-treat and per-protocol analysis.

**Discussion:**

This study uses novel non-invasive VRET, which may provide a possible alternative treatment for dental anxiety and phobia.

**Trial registration number:**

ISRCTN25824611, Date of registration: 26 October 2015.

## Background

It is estimated that as many as 75 per cent of US adults experience some degree of dental anxiety, from mild to severe [1, 2] and that 50-60 % of individuals suffer from a specific fear of dental procedures and dental related stimuli [3]. Given that dental phobia belongs to the most common phobic conditions in our society, finding a suitable specific non-invasive strategy to reduce dental anxiety and treat dental phobia is both warranted and important.

Exposure based treatment programs are considered as the gold standard in the treatment of specific fears and phobias [4–6], including those related to the dental treatment situation [7], [8, 9]. However, potential drawbacks include: (a) the difficulty for patients to mentally visualize the anxiety inducing threat as in imaginal exposure therapy [10], (b) the unwillingness of patients to face the actual threat in in vivo exposure therapy (IVET) resulting in refusal or termination of therapy accounting for 25 % [11], (c) the failure to achieve clinically significant symptom relief, and return of fear, following exposure therapy [12], (d) the poor availability of psychological services [13] and (e) the high costs [14] involved with this treatment.

Recently, VRET has become a viable alternative for in vivo exposure in the treatment of fears and specific phobias [15] including claustrophobia [16], acrophobia [17], fear of flying [18], and spider phobia [19]. This involves conducting exposure therapy using computer generated Virtual Reality (VR) environments by systematic confrontations of patients with their potentially fear-provoking (i.e., ‘conditioned’) stimuli so that habituation occurs [15, 17]. Compared to IVET, VRET is safer because the patients faces the virtual representation of their threat more gradually in a controlled manner, and at their own pace [10]. As the entire exposure process in VRET is completed under privacy of therapists’ office it may elicit less fear of social embarrassment to the patients [20] . VRET can be repeated, whenever and, for as many times [10] as necessary without incurring any additional costs. In-vivo exposure therapy requires training on the part of therapist and is usually delivered by trained psychologists. Compared to this, administration of VRET may just require a working knowledge of computer operation and basic training to operate the apparatus.

VRET is known to elicit a feeling of being “present” in the virtual environment [15, 17]. This sense of presence is considered to be the essence for the effectiveness of VR [21] and has been found to be an important mediating variable [15] between the VR media and the level of anxiety induced [22]. Presence in VR is measured subjectively with questionnaires or objectively with physiological measures [e.g., heart rate (HR), body posture, skin conductance level] [15, 17]. VRET was found to be able to elicit physiologic responses in individuals with fear of flying phobia (measured by HR and skin-conductance) when exposed to VR flights, and these responses decreased following repeated VR exposure flights [23]. Psychophysiological arousal forms the basis for an effective exposure based therapy [24]. A recent systematic review has suggested that VRET does elicit psychophysiological arousal, thereby rendering it to be a promising treatment modality for treatment of anxiety disorders [25]. However, the effect of VRET on HR is inconclusive because of limited well designed studies [25]. Also, there is no published research examining the role of VRET in causing psychophysiological arousal in anxious dental patients. Further, studies examining the relationship between subjective presence and physiological responses are limited [26].

A recent meta-analysis suggests VRET to be slightly, but significantly, more effective than IVET (in vivo exposure therapy) [27]. Although, VRET provides a powerful means of modifying affect, because of its immersive nature, it has not been tested yet as a therapy for dentally related anxiety or dental phobia. In an effort to answer this question, we will use VRET simulation software for the treatment of dental phobia and test its efficacy in the present study. The primary objective of the present study is to determine the efficacy of VRET versus an informational pamphlet control group in terms of dental trait and state anxiety reductions at 1 week, 3 months and 6 months follow-up among a sample of patients suffering from dental phobia. The secondary objectives are to determine (a) the real-time effect of VRET on the course of participant’s physiological response (HR) on exposure with a series of dentally related cues during therapy and (b) the relation between subjective (i.e., presence) and more objective (i.e., HR) measures during VRET. It is hypothesized that: (a) VRET would result in a significantly reduced level of dental trait anxiety [measured by MDAS (Modified Dental Anxiety Scale) and Dental Fear Survey (DFS)] and state anxiety [indexed by VAS-A (Visual Analogue Scale-Anxiety)] at 1 week, 3 months and 6 months follow-up compared to the informational pamphlet control group, (b) VRET would result in significantly higher reduced physiological arousal (i.e., HR) to dental anxious stimuli/cues relative to the baseline HR values following therapy, (c) VRET will demonstrate a positive correlation between subjective and objective measures of ‘presence’.

## Methods

### Trial design

**Fig. 1:**
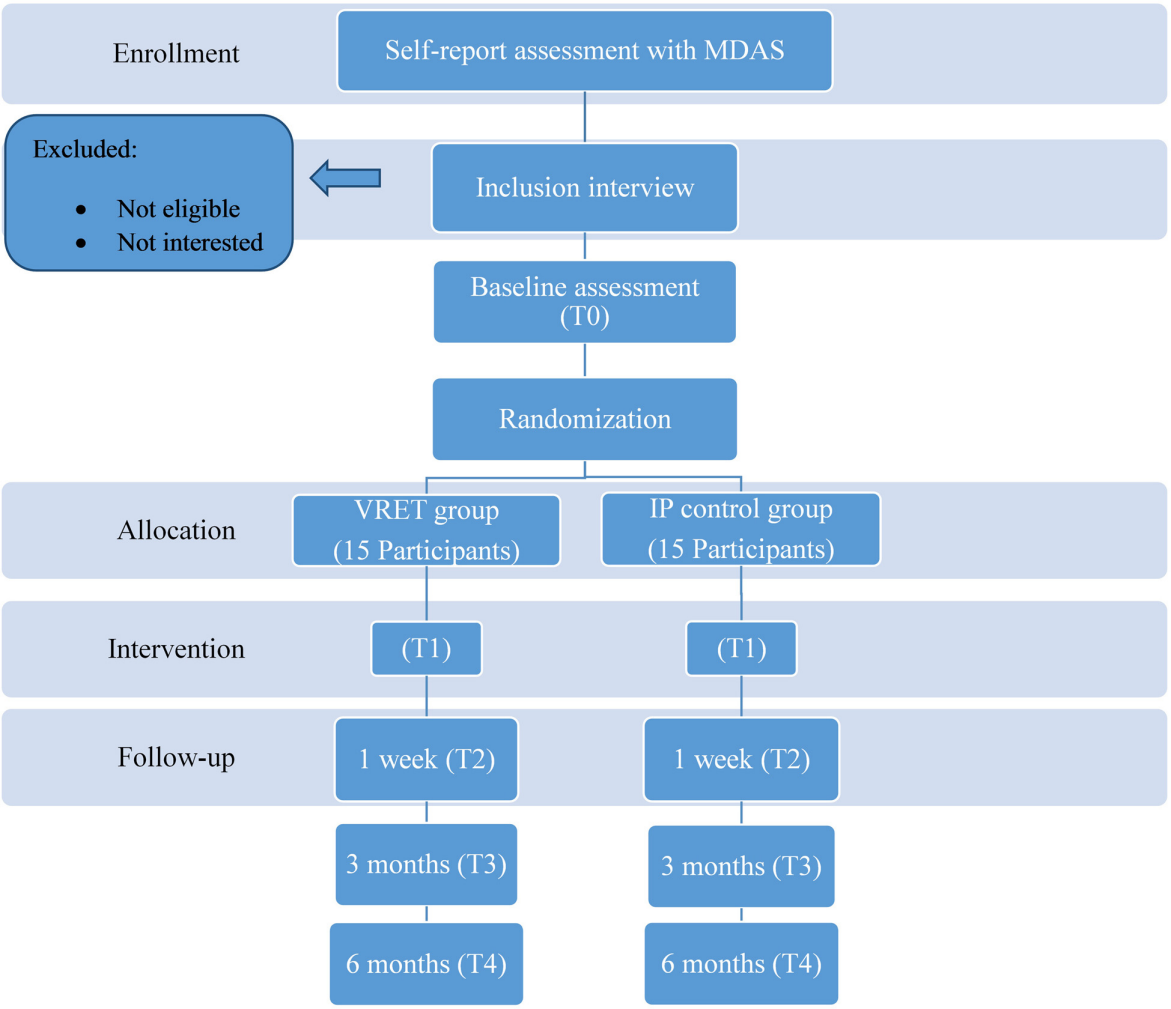
Participant flow and timing of evaluation

The study will be conducted in compliance with local regulations and internationally established principles of the declaration of Helsinki (64^th^ World Medical Association General Assemble, Fortaleza, Brazil, 2013). The study and protocol were approved by the Ethics Commission of the SEGi University (Reference: EC01/14-01). This VRET study will be designed as a single blind (Biostatistician will be blinded), randomized controlled trial with two parallel arms: VRET and Informational Pamphlet (IP) groups with an allocation ratio of 1:1 as shown in Fig. 1. Thirty participants undergo block randomization so that we have equal distribution of participants in both the groups. In order to preserve the allocation concealment, we will be using, sealed, opaque, sequentially numbered envelopes (SNOSE); [28]. Envelopes will be opened serially (next highest number) only after the participant details (patient unique number, date and patient signature) are entered on the envelope. Carbon paper inside the envelope enables transfer of details to the assignment card. Cardboard or aluminum foil inside the envelope renders the envelope impermeable to intense light [29].

The two groups are assessed at baseline (T0), before and after intervention (T1), at 1-week follow-up (T2), 3 months after treatment (T3) and 6 months after treatment (T4) on a set of variables that are linked to the various research questions. The participant flow and timing of evaluation is depicted in Fig. 1.

### Participants

Adult outpatients who have not visited the dentist since last 12 months or those reporting with anxiety and avoidance of dental procedures will be screened and recruited from the outpatient service of Oral Health Centre of faculty of Dentistry, SEGi University at Malaysia. The trial process starts with a short self-report assessment with a MDAS questionnaire to screen for possible dental phobia. Interested participants, who agreed to participate in our VRET Dental Phobia study, with a MDAS score of ≥15, will be contacted and given an appointment by the researcher for an interview. A screening tool, the “Phobia Checklist” [30] will be used for the assessment of dental phobia in this study. This measure for assessing dental phobia has previously been validated against the Structured Clinical Interview for DSM-IV with a sensitivity of 0.95, specificity of 0.99, and an overall hit rate of 97 %. The phobia checklist consists of four questions based on the DSM-IV-TR [31]criteria for specific phobia. The patients are requested to mark either a ‘YES’ or ‘NO’ response to the following questions related to their dental anxiety:

- The sight of the feared object or experiencing the situation evokes an excessive fear response.
- The fear is greater than justified.
- Avoidance or giving up things because of the fear.
- Avoidance of the situation or object causes daily impairment.

An individual is categorized as dental phobic only upon answering ‘YES’ against all four questions of the phobia checklist. In the present study, the checklist will be administered during the screening interview and at 6 month follow-up.

Anticipated timeline for this study will be as follows:

1. Enrollment to Inclusion Interview: Maximum 1 week.
2. Inclusion Interview to Baseline, Randomization & Allocation: Maximum 1 week.
3. Baseline, Randomization & Allocation- Intervention: Maximum 1 week.

### Eligibility criteria

#### Inclusion criteria

Participants should satisfy all criterion for inclusion.

- Meeting the criteria of “Phobia checklist”.
- An age between 18 to 50 yr.
- Any dental phobic patient requiring the following planned dental treatment/s of *at least* 30 minutes per appointment.
  1. Restorative dental procedure which may or may not be requiring local anesthesia.
  2. Extraction procedure requiring local anesthesia.

The participants will be recruited from the out-patient department of the oral health Centre where it is mandatory for every patient to undergo a diagnostic examination. The information about treatment needs will be obtained from the findings of the diagnostic examination.

#### Exclusion criteria

Presence of any criteria mentioned below result in exclusion of the participant.

- Hearing or visual impairment such as stereoscopy blindness or nystagmus.
- Known mental disorders such as psychosis, post-traumatic stress disorder, developmental or intellectual disability and cognitive impairment.
- Known balance disorders such as vertigo and cybersickness.
- Patients with previous history of epileptic seizures.
- Any history of cardiac problems.
- Patients who are undergoing, or have undergone, any cognitive behavioral therapy (CBT)-based intervention for dental phobia.
- Language impediment (cannot understand English).
- Patients wearing glasses of greater than plus 3.5 power.

### Outcome measures

Table 1 and 2 provide an overview of the measures at various time points. Participants of both groups are evaluated using a series of measures as mentioned below. The participants of the VRET group will undergo HR monitoring in real time, assessed for subjective discomfort/distress and evaluated for VR experience at T1 during the intervention as depicted in Table 1 and 2.

**Table 1:** Showing overview of measures at different time periods

		Measurements						
		Primary outcome				Secondary outcomes (Only with VRET)		
		State anxiety (VAS-A)	Trait anxiety (MDAS)	Dental Fear Survey(DFS)	Behavioral test	Physiological parameter (HR)	Subjective Unit of Distress Scale (SUDS)	VR experience
Baseline (T0)	Screening interview	X	X	X	-	-	-	-
Intervention (T1)	Preoperative interview	X	X	X	X	-	-	-
	VRET^a^ session or IP^b^	-	-	-	-	X	X	X
	Postoperative interview	X	X	X	X	-	-	-
Follow-up (T2,T3)	E mail	X	X	X	-	-	-	-
Follow-up (T4)	E mail	X	X	X	-	-	-	-

Note: ‘X’ Indicates timing of measurement of the outcome measures

^a^VRET: Virtual Reality Exposure Therapy

^b^IP: Informational Pamphlet

**Table 2:** Showing measures during VRET session

Measures		VRET session																											
		Baseline phase (10 min)	Training phase (2 min)	Experimental phase(Sessions repeated p,q,r,s,t times until SUDS score ≤2)																									Immediate Post-therapy phase (10 min)
				Scenario #1 (Idle)					Scenario #2 (Mirror)					Scenario #3 (Syringe)					Scenario #4 (Drill with no sound)					Scenario #5 (Drill with sound)					
1	Number of exposures			1	2	3	4	p	1	2	3	4	q	1	2	3	4	r	1	2	3	4	s	1	2	3	4	t	X
2	Subjective measure(SUDS)			X	X	X	X	X	X	X	X	X	X	X	X	X	X	X	X	X	X	X	X	X	X	X	X	X	X
3	Subjective measure(Presence)			X	-	-	-	-	X	-	-	-	-	X	-	-	-	-	X	-	-	-	-	X	-	-	-	-	X
4	Subjective measure(Realism)			X	-	-	-	-	X	-	-	-	-	X	-	-	-	-	X	-	-	-	-	X	-	-	-	-	X
5	Subjective measure(Nausea)			X	-	-	-	-	X	-	-	-	-	X	-	-	-	-	X	-	-	-	-	X	-	-	-	-	X
7	Objective measure(HR)	X	X	X	X	X	X	X	X	X	X	X	X	X	X	X	X	X	X	X	X	X	X	X	X	X	X	X	X

Note: ‘X’ Indicates timing of measurement of the outcome measures

#### Primary outcome measures

##### Anxiety

Will be determined utilizing VAS-A, MDAS and DFS survey.

- Visual analogue scale for state anxiety (VAS-A) [32]. VAS-A will be recorded by asking the participants to draw a cross mark (X) on a 0-100 mm horizontal scale with the extreme left edge of the scale indicating feeling totally calm and relaxed (0) and the extreme right edge, feeling the worst fear imaginable (100). The measured distance from the left edge of the line to the cross mark placed by the participants to the nearest millimeters provides a quantitative variable that can be used in statistical analysis. The VAS-A has been found to be a simple, sensitive, fast, reliable and valid tool to measure level of state anxiety [33].
- Modified Dental Anxiety Scale [34]. The Modified Dental Anxiety Scale (MDAS; Humphris 1995) is a 5-item scale assessing dental (trait) anxiety. Respondents are asked to rate their level of anxiety across five different scenarios (e.g., anxiety response on a previous day to a prospective dental visit, when in the waiting room, when about to have tooth drilled, teeth scaled and injection to the gums) on a 5-point Likert scale ranging from “not at all anxious” to “extremely anxious”, and possible scores range from 5 to 25, with greater scores indicating higher level of dental anxiety. The MDAS shows high levels of internal consistency and good construct validity [35].
- Dental Fear Survey [36]. The Dental Fear Survey (DFS; Kleinknecht 1973) is a 20-item measure used to identify emotional and physiological reactions associated with several aspects of dentistry, as well as avoidance of dental care due to anxiety. Possible scores range from 20 to 100, with greater scores indicating higher levels of dental anxiety. The DFS has established reliability, validity and sensitivity [37].

##### Behavioral avoidance test

A Behavioral avoidance test will be done prior to, and immediately after, the interventions at T1 by means of standardized observation of behaviour, and an interview for both the groups. The test is similar to the VRET scenarios, and provides a baseline behavioral assessment measure to compare the responses of the patient before and after the VRET. This in vivo test represents an observer-rated instrument earlier used by Doering et al. [38]. It contains 5 situations that occur during a dental visit (e.g., sitting in the dental chair, inspection of the oral cavity using dental mirrors, approaching dental syringe, approaching dental drill without sound and approaching dental drill with sound). While the patient undergoes the dental visit it is observed whether he/she is able to tolerate the situation, and he/she is asked by the observer to assess his/her level of anxiety on a scale of 0–10 in each situation. Both observation and answers to the standardized questions are recorded during the procedure.

#### Secondary outcome measures

- Psychophysiological parameter: A heart rate wrist band will be used to record the real time response to VRET at T1 during VRET session. The device will be integrated with the VR software and the output will be recorded during therapy.
- Subjective Units of Distress (SUD) scale: The SUD scale is an eleven-point scale [39] to measure the intensity of subjective distress currently experienced by an individual. It is used by the therapist in desensitization based therapies as a standard to evaluate the progress of the therapy. Typically the patient is asked by the therapist “On a scale of 0-10 where, 0 is no discomfort and 10 is the worst, how do you feel right now”. The researcher will record the SUDS scores during each VR scenario exposure of the VRET session. During the VRET session each VR exposure is repeated, until there is habituation demonstrated by a relatively low SUD score (≤2), and the next scenario is introduced. The total number of VR exposures with each VR scenario that was required to achieve SUDS score of ≤2 will be summed up and recorded by the researcher post-therapy.
- VR experience: will be evaluated at T1.
  Time perception is determined by asking the participants to calculate approximately the duration of VR immersion with therapy [40] and the ratio of subjective and objective duration will be evaluated.
  Presence will be measured using an 11-point verbal rating scale (VRS) [41].
  Realism will be indexed with an 11-point (VRS) [41].
  Severity of nausea (‘cybersickness’) will be measured using an 11-point VRS [41].
  Intention to use VR goggles again will be indexed with a yes/no response.
  Intention to revisit the dental surgery will be measured with a yes/no response.

### Procedure

After arrival at the dental office, participants will receive a detailed explanation about the study from the researcher. All participants will be asked for a written informed consent to participate in the study. The participants will be interviewed to screen for dental phobia using the “Phobia checklist” by the researcher. The interview will take 0.5 to 1.5 hours. The participants will undergo the baseline assessments (T0) which will include use of questionnaires to record the participants’ characteristics such as demographic information, self-reported oral health, self-reported dental attendance and history of a bad experience with a previous dentist [42]. Completing the questionnaires will take 10-15 minutes. The interview and questionnaire are completed on the same day. Informed consent and pre-operative data will be collected in the waiting area. The participants undergo VRET at their own pace free of cost. The assessment of state and trait dental anxiety will be done utilizing the VAS-A [32], MDAS [34] and DFS [36]. After T0, the participants will be block randomized as mentioned in Fig. 1 into VRET intervention and informational pamphlet control groups.

### VRET group

#### Description of the VRET system used

The VRET software has been developed for this study by Virtual Simulations Inc in collaboration with KR and ADJ. The hardware comprises of two networked computers of which the VR-simulator PC (Personal Computer) renders the virtual environment and the other User interface-PC (UI-PC) allows the therapist to control and individualize stimuli presented. The VR-simulator software will generate the VR dental environment using a Dell XPS-8700 desktop with 4th Generation Intel Core i7-4790 processor (8 M Cache, up to 4.0 GHz) and ASUS NVIDIA GEFORCE GTX 750 TI OC 2GB GDDR5 graphic card. The system will generate the display at a rate of 60 frames per second. To immerse the participants in the VR dental environment we will use an Oculus development kit 2 HMD (Head Mounted Display) with a resolution of 960X1080 per eye and with a 100 degree field of view (nominal).

#### Treatment room configuration

The waiting and VR treatment area will be shaped, using cues usually present in these areas. For VRET, a simulated dental environment will be created, with a dental chair, overhead light, dental instruments and to enhance immersion characteristic dental clinic-related smell (drops of oil of cloves on cotton wool) will be introduced. The room temperature will be maintained at 21 °C.

The treatment room where the study will be conducted will be equipped with:

1. Two networked computers and a HMD as described above.
2. Oculus positional tracking camera mounted on a tri-pod: To track the head movements in real-time.
3. Logitech C-920C webcam mounted on a tri-pod: To record the video for future validations and evaluate the participant’s body response during VRET.
4. YOGA Mini Tie Clip Condenser Microphone: To record the voice of the participants during VRET, It is synchronized with the webcam.
5. MIO LINK HR wrist band: To record the real-time HR of the participants during therapy.
6. SUUNTO movestick mini: For wireless transfer of HR data from the wrist band to the simulator PC.
7. PHILIPS wireless portable speaker BT100B/37: To produce characteristic dental drill sound.

#### The simulated dental experience

The VRET system will be operated by the author of this study. Training will be provided by a clinical psychologist and Virtual Simulations Inc. When participants assigned for VRET visit the dental office the researcher records the unique reference number and initiates the VRET session. The Simulator PC, UI PC and video camera are turned on. Participants will be seated in a supine position comfortably in the dental chair and will be assisted in wearing the HMD (in OFF mode) and HR bracelet (left hand) by the researcher. Also, a hand held computer mouse will be given to the participants which acts as a panic button. The participants will hold the computer mouse with their right hand and will be instructed how to activate the panic button by clicking the mouse (under their control) when the presented scenario feels unbearable to them during the VRET session. The simulator PC is connected with the UI PC. On the UI window the researcher enters the patient/subject name, connects the UI with VR simulator by entering VR Internet Protocol address and presses the start button of the log record for real time HR recording continuously during the session.

#### Phases of VRET session: The phases are depicted in Table 2

##### Baseline phase

During this phase, the participants will see no display (Black screen) through the HMD (In OFF mode) for 10 minutes. Concurrently, the VR simulator software will record the HR. The purpose will be to record the resting HR as per HR wrist band manufacturer’s instructions.

##### Training phase

The HMD is turned ON once during the entire VRET session at this phase. In general, participants may show a higher physiological response when exposed to see something novel, this is referred to as orientation effects [43]. To overcome this, the participants in this phase will first view a surrounding 3D (3 Dimensional) stereoscopic scene of simulated dental environment through the HMD and only take a passive role by watching the interactive scene for 2 minutes. The participants are encouraged to turn his head and look around the virtual dentist’s office by way of the built-in motion tracking present in the HMD. Thus, the test subject will be lying on a real dentist chair while he will see its virtual counterpart inside the HMD as he looks around, turning his head. To allow the test subject to feel immersed in the virtual environment, we will include a generic 3D model of a person lying on the dentist chair such that when looking down, the subject will see what feels like his own body. The virtual environment seen by the patient will be displayed to the researcher on the computer screen and the HR will also be recorded.

##### Experimental phase

In this phase, participants are exposed to five different VR scenarios (Idle, Mirror, Syringe, Drill with no sound, Drill with sound). The duration of each exposure will be 35 seconds. The interactive part of the simulated dental environment will be controlled by the researcher using the tool selection option on the UI window of the computer. The researcher will be able to control the playback of the VR scenarios.

VRET is conducted using a pre-determined hierarchy as follows:

1. Scenario #1 (Idle). This shows a dental operatory with various instruments surrounding the patient’s chair and a virtual dentist sitting next to the patient’s right hand inside the 3D scene.
2. Scenario #2 (Mirror). This shows the virtual dentist inspecting the oral cavity by picking the dentist’s mirror from the tray and approaching towards the patient’s oral cavity.
3. Scenario #3 (Syringe). This shows the virtual dentist performing injection by picking the syringe from the tray and approaching towards the patient’s oral cavity.
4. Scenario #4 (Drill with no sound). This shows the virtual dentist picking the drill and approaching towards the patient’s oral cavity with no sound of the drill.
5. Scenario #5 (Drill with sound). This shows the virtual dentist picking the drill. The drill makes the characteristic dental drill sound when approaching towards the patient’s oral cavity.

Participants will be exposed to the virtual dental scenarios in a gradual manner. To give the participants a gradual and optimal VRET, participants will be encouraged to rate their anxiety every 35 seconds following immersion with each VR scenario exposure by means of SUDS [39]. The general rule applied during exposure therapy is that the exposure is continued until the subjective score of the patient is reduced to less than 50 % [44].The VRET which we are planning to use in the current experiment has only a limited set of stimuli. Accordingly, we have decided to have a safe cut off of ≤2 SUDS scores before moving to the next VR scenario. This criterion was earlier applied in a similar study [17]. The exposures with scenario 1, 2, 3, 4 and 5 are repeated p,q,r,s and t times respectively until SUDS score of ≤2 is achieved. For instance, when participant reaches ≤2 SUDS with VR scenario of sitting idle on the dental chair then the next VR scenario of inspecting the oral cavity with a dental mirror is introduced. In subsequent sessions, the intensity of VR experiences will be progressively increased from a less threatening scenario to a more threatening scenario to make the simulation more realistic while focusing on areas of particular stress. For example, if a dental phobic participant report a fear of drills, the exposure is progressively increased from Scenario-1 (less fear evoking –‘Sitting in the dental chair’) to Scenario-5 (more fear evoking –‘Drill with sound’). To this end, situational cues that are simulated with VR scenarios are presented by the therapist in a well-controlled manner. During the simulated dental procedure participants will be asked to keep their mouth open similar to real dental experience from Scenario #2 (Examination with mirrors) through Scenario #5 (Drill with sound) and are advised to follow the instructions of the researcher, for example ‘to open their mouth really wide’. The added stimulus such as keeping the mouth open will be standardized and applied to all the participants. Participants will receive the therapy in one long session for 150 minutes with a 10-min break/s to avoid simulator sickness. If the participant’s SUDS rating has not decreased to 2 with any of the VR scenarios within 30 minutes of exposure the researcher will continue with the next VR scenario. The session will be video recorded for future validations and analysis of the subject’s body response during the VRET exposure.

Only verbal guidance and exposure techniques will be used on the participants during VRET. There will be no relaxation or other CBT-based interventions during therapy. Participants will be instructed to become as involved as possible and focus on their most frightening stimuli of the particular part of the virtual environment (describing the situation, any strange sensations and their feelings). This will be done to avoid dissociation from the VR experience. During the simulated treatment, real-time recording of the psychophysiological parameters (HR) will be determined to obtain more ‘objective’ measures of the physiological state of the participants during therapy. The HR responses obtained with different cues (Idle, Mirror, Syringe, Drill with no sound and Drill with sound) in this phase will be compared with the baseline phase (No display) to study the HR variations. The subjective measures of presence, realism and nausea (cybersickness) are recorded following the 1^st^ exposure of each VR scenario as shown in Table 2.

##### Immediate post-therapy phase

In this phase, the HR is recorded for 10 minutes and the participants are asked to fill out a series of self-reported measures as displayed in Table 2 and undergo the Behavioral Avoidance Test. The total number of VR exposures with each VR scenario will be summed up and recorded by the researcher. Also, the participants fill out the questionnaire on over-all presence, realism and nausea (cybersickness). After the VRET session, participants will remain in the waiting room for 15 min before leaving. This will be done in order to make sure that are no negative side effect of VRET *a posteriori*.

One week after treatment and at intervals of 3 and 6 months, the study participants will receive questionnaires that are used to assess the participants’ current dental anxiety scores and avoidance tendency (see Table 1) by email.

### Informational pamphlet control group (IP)

Participants in the IP group will be given three pages of information based on that of a dental website company, and of a fact sheet about dental anxiety from the Academy of General Dentistry [45]. The pamphlet will contain details about the standards of care such as patient comfort, description of dental procedures and postoperative pain management. Participants will be seated in the same area where they completed the screening and are given time to review the pamphlet in detail. Also, an opportunity is given to the participants to ask the researcher information about dental phobia. The study participants will receive a total of 5 assessments, T0, T1, T2, T3 and T4 similar to VRET intervention group.

Following interventions, the participants of both the groups are told that if they have any question/s about their condition, they can call a telephone helpline that facilitates patient access to information and address any questions they might have (between 08:00 to 17:00 from Monday to Friday).

## Sample size

Since the approach of the study is novel, there is scanty comparable research available to use for a sample size calculation. We used the difference between two independent means’ for sample estimation (independent-samples t-test). In a previous study [46] the response within each subject group was normally distributed with standard deviation (MDAS) 4.22. If the true difference in the experimental and control means (MDAS) is 6, we will need to study 12 experimental subjects and 12 control subjects to be able to reject the null hypothesis that the population means of the experimental and control groups are equal with probability (power) 0.9. The Type I error probability associated with this test of this null hypothesis is 0.05. Since drop-outs are unavoidable when collecting follow-up data, the sample number needs to be adjusted for the estimated drop-out rate. We are estimating a drop-out rate of 15 % in this study. Adjusting the sample for 15 % drop-out and 10 % for VR crash results in sample of 30 participants (15 experimental and 15 control participants).

## Data analysis

The analyses will be performed based on the Consolidated Standards of Reporting Trials (CONSORT) statement regarding e-health [47]. An independent biostatistician will carry out the statistical analysis using SPSS 20.0.The recorded data will be labelled so that the biostatistician will not be able to distinguish between the two groups. All data will be analyzed using an intention-to-treat analysis. We will also conduct a per protocol analyses. Descriptive statistics will be calculated, whereas discrete variables will be summarized by frequencies or proportions. Continuous variables will be reported as means and standard deviations or medians and range (depending on the measurement level of the variables). Data will be checked for differences at T0, T1, T2, T3 and T4 between the VRET and informational pamphlet group using independent samples t-test. Between-group changes at post treatment will be calculated by using a 2 (pre-treatment/post-treatment) × 2 (VRET/Informational pamphlet) repeated measures MANOVA. Post hoc comparisons will be applied if clarification of the main effects of the MANOVA is needed. Effect sizes between the groups will be calculated with Hedges’ g. Clinical significance change analyses as described by Jacobson and Truax [48] will be conducted to determine whether the changes from pre-test to post-test are clinically significant [48]. Missing data analysis will be performed and the reason for non-adherence will be registered.

The continuous monitoring of HR data will help us determine whether the physiological changes diminish following multiple exposures with each scenario (suggestive of habituation-Reliability of therapy).

To evaluate the correlation between subjective measures of VR experience (presence, realism, nausea) with objective measure of HR during each VR scenario, only the first exposure measures will be considered to eliminate the order effects as shown in Table 2.

Since the baseline physiology levels will vary widely between individuals, we will be determining the percentage change from baseline HR during the first exposure of each VR scenario for analyses rather than considering the absolute values. The percentage change of heart rate (%HR) will be calculated as follows:

$$ \%\mathrm{H}\mathrm{R} = \left(\mathrm{MeanVR} - \mathrm{MeanBaseline}\right)/\mathrm{MeanBaseline}, \times 100 $$

where,

MeanVR: Mean of HR during the VRET of 1^st^ exposure of each VR scenario, and

Mean Baseline: Mean of HR during baseline phase of VRET session.

This will help us to compare and correlate between:

1. Self-reported subjective scores (SUDS) and Objective scores (%HR during first VR exposure of each scenario) of anxiety. Determines the validity of HR (how well do self-report scores correlate with physiological measure of HR).
2. Subjective measures of presence and the objective scores (%HR).
3. Subjective measures of presence and realism.
4. Objective scores (%HR) and subjective realism measures.
5. Objective scores (%HR) and subjective cybersickness (nausea) measures.

The association between measures will be determined using the Pearson correlation coefficient.

## Discussion

The design of this randomized clinical trial offers pathways to address the efficacy of VRET in the treatment of long term trait dental anxiety. Therefore, we developed (KR, ADJ and MB) a VRET device based on exposure therapy models and will be testing the efficacy of this intervention among people with dental phobia immediately after therapy, 1 week, 3 months and 6 months post therapy, and will be comparing with the results of an informational pamphlet intervention.

In recent years VRET has become a viable treatment alternative for in vivo exposure therapy as evidence based treatment for specific phobias with the advantage of allowing patients to face their fears in a controlled and thus safe, environment. Patients may not feel the anticipatory fear of getting hurt as they are aware that the VRET simulation is purely virtual and that these situations can be stopped, paused, restarted as well as repeated, whenever, for as many times as deemed necessary [10]. Accordingly, patients’ acceptance of VRET could be relatively high.

Another important advantage of the application of VRET for dental phobia is that it may not require specialized training and the entire exposure process can be completed by a computer in the safety and privacy of the practitioner’s office [10]. To this end, the study is a feasibility study. If proved successful, the VRET device can be rendered by any dental auxiliaries in a general dental practice which could make it a cost-effective solution. For example, because VR technology has applications in telemedicine, in which diagnosis and treatment can be administered from a distant site [49]. This may make VRET applicable in rural dental clinics, in which the therapist might not be available at long distances and the therapist views on a monitor with which the subject is interacting, and comment appropriately and therapeutically.

The strengths of our study design include randomized group allocation, use of validated standardized assessments and a long term follow-up. A limitation of the present study is the absence of IVET as gold standard control group. Yet, it is hoped that the findings of this study will provide evidence in support of the efficacy of VRET and therefore will be an important step in the treatment of dental phobia in the setting of the dental practice.

## Abbreviations

3D: 3 Dimensional
CBT: cognitive behavioural therapy
CONSORT: consolidated standards of reporting Trials
DSM: diagnostic and statistical manual of mental disorders
DFS: dental fear survey
HMD: head mounted display
HR: heart rate
IP: informational pamphlet control group
IVET: in vivo exposure therapy
MANOVA: multivariate analysis of variance
MDAS: modified dental anxiety scale
PC: personal computer
RCT: randomized controlled trials
SUDS: subjective unit for distress/disturbance scale
UI: user-interface
VAS-A: visual analogue scale for anxiety
VR: virtual reality
VRET: virtual reality exposure therapy
VRS: verbal rating scale
